# Variability in summer surface residence time within a West Antarctic Peninsula biological hotspot

**DOI:** 10.1098/rsta.2017.0165

**Published:** 2018-05-14

**Authors:** Josh T. Kohut, Peter Winsor, Hank Statscewich, Matthew J. Oliver, Erick Fredj, Nicole Couto, Kim Bernard, William Fraser

**Affiliations:** 1 Department of Marine and Coastal Sciences, Rutgers, The State University of New Jersey, 71 Dudley Road, New Brunswick, NJ 08901, USA; 2 College of Fisheries and Ocean Sciences, University of Alaska, Fairbanks, 2150 Koyukuk Dr., Suite 245 O'Neill Bldg., Fairbanks, AK 99775-7220, USA; 3 College of Earth, Ocean and Environment, University of Delaware, 700 Pilottown Road, Lewes, DE 19958, USA; 4 Computer Science Department, The Jerusalem College of Technology, 21 Havaad Haleumi St., PO Box 16031, Jerusalem 91160, Israel; 5 Scripps Institution of Oceanography, University of California, San Diego, 9500 Gilman Drive #0213, La Jolla, CA 92093, USA; 6 College of Earth, Ocean, and Atmospheric Sciences, Oregon State University, 104 CEOAS Admin Bldg, Corvallis, OR 97330, USA; 7 Polar Oceans Research Group, PO Box 368, Sheridan, MT 59749, USA

**Keywords:** biological hotspot function, residence time, Lagrangian particle trajectories, high-frequency radar, West Antarctic Peninsula, circulation

## Abstract

Palmer Deep canyon along the central West Antarctic Peninsula is known to have higher phytoplankton biomass than the surrounding non-canyon regions, but the circulation mechanisms that transport and locally concentrate phytoplankton and Antarctic krill, potentially increasing prey availability to upper-trophic-level predators such as penguins and cetaceans, are currently unknown. We deployed a three-site high-frequency radar network that provided hourly surface circulation maps over the Palmer Deep hotspot. A series of particle release experiments were used to estimate surface residence time and connectivity across the canyon. The majority of residence times fell between 1.0 and 3.5 days, with a mean of 2 days and a maximum of 5 days. We found a highly significant negative relationship between wind speed and residence time. Our residence time analysis indicates that the elevated phytoplankton biomass over the central canyon is transported into and out of the hotspot on time scales much shorter than the observed phytoplankton growth rate, suggesting that the canyon may not act as an incubator of phytoplankton productivity as previously suggested. It may instead serve more as a conveyor belt of phytoplankton biomass produced elsewhere, continually replenishing the phytoplankton biomass for the local Antarctic krill community, which in turn supports numerous top predators.

This article is part of the theme issue ‘The marine system of the West Antarctic Peninsula: status and strategy for progress in a region of rapid change’.

## 1. Introduction

In the coastal West Antarctic Peninsula (WAP), the food web is comparatively short and characterized by intense phytoplankton blooms that are grazed by Antarctic krill *(Euphausia superba*)*,* a primary prey source for penguins and other predators. Although krill aggregations occur throughout the WAP [1], the distribution of penguin populations and their associated foraging areas are spatially coherent with submarine canyons and near-shore deep bathymetry within biological hotspots that are characterized by enhanced biological production and elevated biomass [2–5]. Within these hotspots, penguin foraging locations may be highly variable [6] in accordance with the small-scale patchy distribution of their prey [7,8]. However, the spatio-temporal coherence between penguin colonies and these deep canyons suggests that resources are transported to, and concentrated within, these hotspots. The implication is that circulation features associated with these canyons may enhance food web transfer, termed ‘trophic focusing' by Genin [9], and are the underlying physical mechanism that maintains the hotspot.

**Figure 1.**
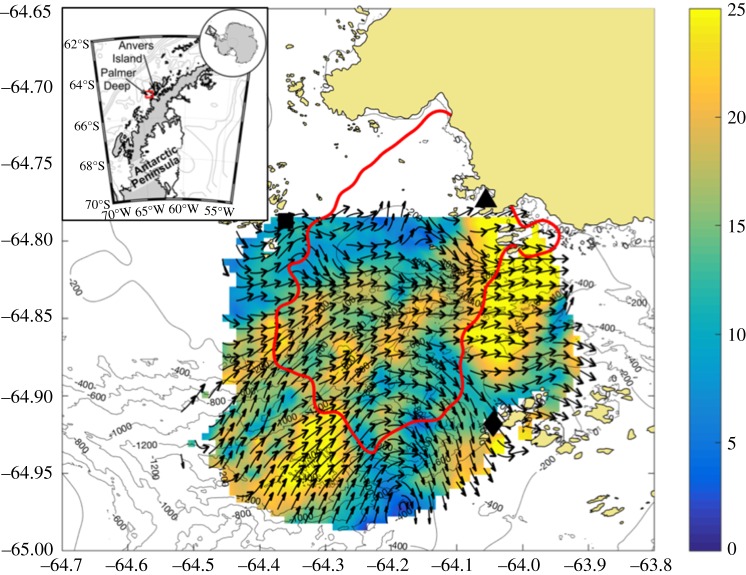
Map of study site with hourly map of surface currents (cm s^−1^) sampled on 30 January 2015 00:00 GMT. The HF radar sites at Palmer Station (black triangle), Joubin Islands (black square) and Wauwermans Islands (black diamond) are also shown. The red line is 99.5% contour for tagged Adélie penguin locations from 2002–2011 [6].

Palmer Deep canyon is a representative biological hotspot located near Palmer Station, Anvers Island, along the WAP (figure 1). Here, local islands have been occupied by Adélie penguins *(Pygoscelis adeliae)* for nearly 1000 years [10] and now include growing gentoo *(P. papua)* and chinstrap (*P. antarctica*) penguin colonies, suggesting Palmer Deep is conducive to penguins in general. Despite variation in climate over the last 1000 years [11], the persistence of these penguin colonies suggests that the presence of the canyon mediates and/or enhances the accessibility and predictability of their prey over ecological time scales [2]. Palmer Deep has higher phytoplankton biomass than the surrounding non-canyon regions [5], but the circulation mechanisms that transport and locally concentrate phytoplankton and attract Antarctic krill, potentially increasing prey availability to penguins, are unknown. Prior work in the region suggests that local upwelling supports and maintains local phytoplankton growth that in turn fuels the biological hotspot [12]. This would require that phytoplankton blooms are retained within the hotspot, benefiting from upwelled nutrients from below. Therefore, surface circulation patterns within the hotspot itself are critical to understanding the mechanisms that fuel and maintain the primary productivity that supports the food web. Traditionally, circulation associated with canyons is inferred from moorings and ship-based surveys [13–15]. Additionally, numerical modelling simulations of the three-dimensional flow field have been used to estimate the fate of simulated passive particles [16,17]. Specifically, Lagrangian particle tracking experiments have been used in the WAP to study transport pathways between the outer shelf and near-shore biological hotspots [18], connectivity between different regions of the WAP [19], and the transport and fate of larval Antarctic krill along the WAP [20,21]. These studies identify key transport pathways across the shelf that link the Antarctic Circumpolar Current offshore to the near-shore biological hotspots. The models used for these experiments cover the entire shelf region with horizontal resolutions down to approximately 4 km [18,19]. Additionally, particle tracking experiments have been used to quantify the residence time based on the loss of deployed particles over time [19]. Longer residence times may be indicative of biological hotspots acting as biological incubators that support local phytoplankton growth. While these modelling studies provide valuable insight into the role transport may have on the ecology of the WAP, they typically do not resolve local processes that may influence the availability of phytoplankton to upper trophic levels within these hotspots. New observational tools resolve these circulation processes at the scale of the hotspot itself. Ocean observing technologies such as autonomous underwater vehicles and high-frequency radar (HFR) can augment existing observations to better resolve circulation within the hotspot [22].

Here we discuss the residence time of the surface layer over the Palmer Deep hotspot using passive particle trajectories estimated from HFR surface current maps. Unlike prior work, we use observed currents, mapped at high temporal (hourly) and spatial (1 km, horizontally) resolution, over the entire canyon. We focus our analysis on a single summer foraging season of the local Adélie penguin colonies (January–February 2015).

## 2. Methods

### (a) High-frequency radar

HFR systems, deployed along a coastline, use Bragg peaks within a transmitted signal (3–30 MHz) scattered off the ocean surface to calculate radial components of the surface velocity at a given location [23]. Individual sites, composed of a transmitting and receiving antenna, generate maps of surface component vectors directed towards the antenna with range resolution of 500 m radially and 5° in azimuth. Since these data are based on the return scattered off surface gravity waves, the observations are representative of the circulation at water depths that influence the surface waves, termed effective depth [24]. Operating at central frequencies of 25 and 13 MHz, our network measured the circulation at an effective depth of 0.5 m [24]. While the HFR provides a highly resolved surface current field, it should be noted that these horizontal velocity fields are influenced by vertical velocities associated with mesoscale features (i.e. upwelling, downwelling) but do not resolve the scales of vertical turbulence associated with winds, moving ice, surface heating/cooling or brine rejection. An analysis of 10 years of autonomous glider hydrographic surveys in the region shows that, during the summer months, a homogeneous surface mixed layer forms and that phytoplankton blooms were found to be distributed evenly throughout this layer [25]. Other HFR deployments sampling similar homogeneous surface mixed layers have shown that, due to the low shear within the surface layer, the circulation measured at the effective depth is representative of currents deeper into the surface mixed layer [26]. This suggests that our HFR observations are representative of the horizontal circulation influencing the phytoplankton biomass within the homogeneous surface mixed layer.

In November 2014, we deployed the three-site HFR network to provide coverage of Palmer Deep canyon and the surrounding flanks. The data footprint covered the historic range of Adélie penguin foraging with hourly surface current maps that resolved the surface circulation dynamics influencing the transport of phytoplankton within the surface layer (figure 1). The first HFR site was deployed at, and powered by, Palmer Station. The other two sites were deployed at the Joubin and Wauwermans Island chains (figure 1), and relied on remote power modules (RPMs) that were constructed on site. The RPMs generated the required power for the HFRs through a combination of small-scale micro wind turbines and a photovoltaic array with a 96 h battery back-up [22]. The RPMs consisted of a single watertight enclosure, used to house power distribution equipment, the HFR, and the communication gear. Built-in redundancies within the RPMs, including wind and solar energy harvesting, and independent battery banks, ensured that, should any one component fail, the unit would be able to adjust autonomously. Direct communication between the two remote sites and Palmer Station was enabled with line-of-sight radio modems (900 MHz Freewave), which enabled remote site diagnostics, maintenance and surface current data transmission in real time.

The three-site network collected hourly measurements of ocean surface currents over our two-month study period, which covered the local Adélie penguin breeding season. Every hour, radial components from each of the three sites were geometrically combined into two-dimensional vector maps using an optimal interpolation algorithm [27]. The total vector maps were calculated on a fixed 1 km grid covering an approximately 1500 km^2^ area of ocean over the region of Palmer Deep (figure 1). The average data coverage was 97% during our months of interest between 1 January 2015 and 1 March 2015. The raw surface velocity fields were post-processed to remove the local tides using the Matlab software toolbox, t-tide [28]. The tidal constituents were fitted to the raw surface currents measured between 1 December 2014 and 1 May 2015 (151 days) at each point in the HFR grid. Thirty-five different tidal constituents, within the 95% confidence interval (CI), accounted for 12.3% of the total variance of the raw velocities. The four most energetic tidal constituents in the diurnal and semi-diurnal bands were the (O1, K1) and (M2, S2), respectively. The de-tided velocity time series at each HFR grid point were calculated as the measured raw velocity minus the tidal fit. All other high-frequency variability not associated with tides was retained in the de-tided data. Maps of both the raw and de-tided surface currents were used to estimate the time evolution of the residence time of the surface layer with and without the effect of tide.

### (b) Simulated particle release experiments

**Figure 2.**
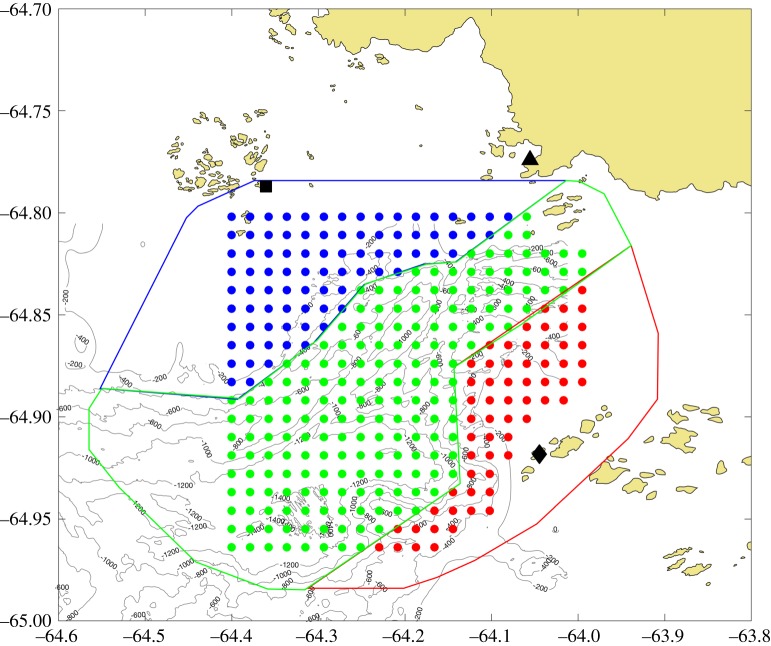
Map of the simulated drifter release points for the central canyon (green), JIF (blue) and WIF (red) regions. The sub-regions for the connectivity analysis are also contoured in the same respective colours. The HFR sites are indicated as in figure 1.

A series of particle release experiments were used to estimate residence time over the two-month study period. Raw and de-tided hourly surface current maps provided by the HFR network were used to simulate passive particle trajectories initially released on a fixed 1 × 1 km grid (matching the resolution of the underlying HFR data) centred over the Palmer Deep canyon (figure 2). For each particle release experiment, one passive particle from each grid point (332 particles in total) was released and advected in the HFR velocity field with a fourth-order Runge–Kutta integration scheme. The position of each particle was tracked hourly until it reached the edge of the domain (figure 2). These experiments were repeated every 6 h for the raw and de-tided velocity fields from the first release on 1 January 2015 00:00 GMT until the last on 1 March 2015 00:00 GMT.

Residence time was estimated from the particle trajectories following the methods described in [19]. Briefly, residence time for a given release experiment was based on the e-folding time scale or the time when the fraction of particles that remain within the HFR footprint was reduced by the first e-folding scale, which is 36.79% of the number initially released. With this method, we estimated a residence time based on the raw and de-tided velocity fields for each 6 h release experiment throughout our two-month study.

We also estimated residence time within, and connectivity between, sub-regions within the HFR footprint (figure 2). Three regions were defined within the hotspot based on the underlying canyon bathymetry, including the central canyon (CAN), Joubin Islands Flank (JIF) and Wauwermans Islands Flank (WIF). The particle grid included 187, 84 and 61 particles for the CAN, JIF and WIF sub-regions, respectively (figure 2). For these experiments, the residence time was estimated as the time required to reduce the fraction of particles that remain in the HFR footprint to one e-folding scale (36.79%) of those initially released in each sub-region. For example, at a given release time, the residence time for JIF was the time it took for 53 of the 84 particles to leave the HFR footprint.

Tracks of particles released from each sub-region were also used to quantify the connectivity between the sub-regions. The paths of each particle released from each point on the grid were tracked every hour until they left the HFR footprint. These tracks were then used to determine the percentage of particles initially released from each sub-region that entered either of the other two sub-regions. This percentage was calculated for each release experiment, every 6 h throughout the two-month study.

### (c) Meteorological data

Meteorological data were collected at the two remote HFR sites in the Joubin and Wauwermans Islands (figure 1). These data included 15 min measurements of air temperature, wind and solar radiation. The winds over the HFR footprint were estimated as the average of the winds measured at the Joubin and Wauwermans Islands sites. For this analysis, we refer to the two-site average winds as merged winds. For comparison to the residence time estimates, the 15 min merged wind fields were averaged to match the residence time estimates for each 6 h release. For example, the residence time estimated from the particles released on 1 January 2015 and 00:00Z was 1.9 days. Therefore, the merged wind field matched to this residence time was averaged from the initial release at 00:00Z on 1 January 2105 through 21:45 on 2 January 2015, 1.9 days later. This was repeated for each 6 h particle release experiment to average the winds over the same time that the particles transited through the HFR field.

## 3. Results

### (a) Residence time

The basic statistics of the residence time estimated for the entire domain and each sub-region are shown in table 1. Overall there were small differences between the residence times estimated from the raw and de-tided velocity fields. The mean residence time for both the raw and de-tided currents over the entire domain was 2.1 ± 0.9 days. The similarity between the raw and de-tided results indicates that the sub-regions are of sufficient area to ensure that the particles are not immediately removed by the excursion of a single tidal cycle. The majority of residence times over the entire domain fell between 1.0 and 3.5 days, with a few outliers extending out to a maximum of 5 days. Of all the sub-regions, the central canyon most closely matched the entire domain, with values primarily between 1.0 and 3.2 days. The JIF generally had longer residence times, with a majority of values falling between 1.0 and 4 days, and a few outliers out to the maximum observed residence time of 7.8 days. The WIF fell on the opposite side of the distribution, with most residence times between 0.2 and 2 days. The minimum residence time was shorter in the WIF sub-region compared to all the other regions. In summary, all regions indicate that residence times are typically less than 4 days, with a mean of approximately 2 days. The JIF and WIF fall on the longer and shorter side of this general distribution, respectively. Given the small difference between the residence times estimated from the raw and de-tided velocity fields, the remainder of the analysis focused on the particle trajectories based on the raw velocities.

**Table 1.** Residence time for each region.

region	number of particles	velocities	mean residence time (days)	maximum residence time (days)	minimum residence time (days)
entire domain	78 684	raw	2.1 ± 0.9	6.0	0.7
		de-tided	2.1 ± 0.9	5.0	0.7
central canyon	44 319	raw	2.4 ± 1.1	6.8	0.6
		de-tided	2.3 ± 1.1	6.7	0.6
Joubin Islands Flank	19 908	raw	2.5 ± 1.6	7.8	0.3
		de-tided	2.5 ± 1.5	7.7	0.3
Wauwermans Islands Flank	14 457	raw	1.2 ± 0.9	5.0	0.2
		de-tided	1.2 ± 0.8	5.1	0.2

### (b) Connectivity

Connectivity between the different sub-regions was estimated from the individual particle trajectories. Connectivity was quantified as the percentage of particles released from one region that entered into another region over the two-month period. As with residence time, connectivity was estimated for each sub-region relative to the other two for each 6 h release. Basic statistics of the connectivity are shown in table 2. Given the high variability relative to the reported means, we report on the sample mean as a general statistic of the connectivity between sub-regions and discuss possible drivers of this variability in the following sections. For the central canyon, a greater percentage of particles moved southeast over the WIF compared to the JIF (53% compared to 30%). Particles that did not move into either flank exited the canyon to the southwest, or through the canyon head towards the northeast into the Bismarck Strait. While a similar percentage of particles (approx. 25%) exited both ends of the canyon, the entrance to the Bismarck is one-third the width across the southwest canyon exit. Therefore, the particles moving northeast into the Strait are more concentrated compared to those leaving through the offshore exit of the canyon. For particles initially released over both flanks, we found that more particles moved across the canyon from the JIF to the WIF (26%) than from the WIF across the canyon to the JIF (7%). Similarly, most of the particles that entered the canyon originated over the JIF.

**Table 2.** Connectivity between the regions.

initial region	destination region	mean percentage of particles	max percentage of particles	min percentage of particles
central canyon	Joubin Islands Flank	30 ± 20	72	0
	Wauwermans Islands Flank	53 ± 25	100	0
Joubin Islands Flank	central canyon	51 ± 30	98	0
	Wauwermans Islands Flank	26 ± 30	98	0
Wauwermans Islands Flank	central canyon	37 ± 23	98	0
	Joubin Islands Flank	7 ± 14	67	0

### (c) Residence time versus local winds

**Figure 3.**
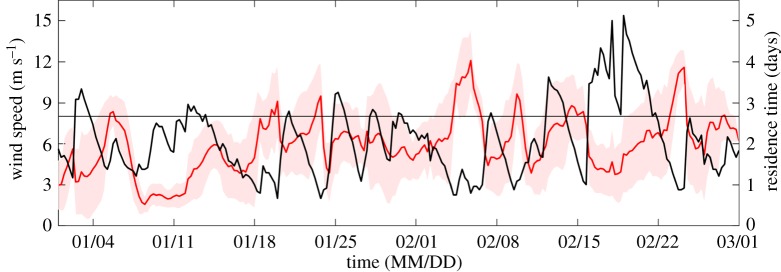
Time series of residence time (black) and average merged wind speed measured at the Wauwermans and Joubin Island sites (red). The standard deviation of the merged winds averaged over each residence time estimate is shaded in red. The horizontal line represents a wind speed of 8 m s^-1^.

The time series of residence time estimated for the entire domain was compared to local winds observed on either side of the canyon. Over the two-month period, the residence time of the entire domain fluctuated between the minimum of 0.6 days and the maximum of 5 days (figure 3). The longer residence times occurred later in the time series towards the latter half of February. Shorter events, with residence times less than 1 day, were scattered throughout the time series. The average wind speed based on estimated residence time fell between 2 and 12 m s^−1^. The calmest winds are observed in mid-January followed by a much more variable wind field in February, with several events greater than 9 m s^−1^ lasting one to several days. Throughout the time series there was a consistent pattern of lower residence times coincident with wind speeds greater than 8 m s^−1^ (horizontal line, figure 3). This was most evident towards the end of the time series where large fluctuations in wind speed were matched with changes in residence time. During these February events, there was a rapid decrease in residence time with increased wind speed followed by a lengthening of residence time as the winds relaxed between events (figure 3). We tested for the effect of wind speed on residence time in two ways. First, we performed a major axis regression (model II) on the wind speeds and residence times [29]. Unlike ordinary least-squares regressions, major axis regression assumes errors in both wind speeds and residence time. We found a highly significant negative relationship 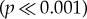 between wind speed and residence time. For every 1 m s^−1^ increase in wind speed, residence time decreased by 0.18 (95% CI −0.19 to −0.24) days. However, there was a large degree of unexplained variance in residence time with respect to wind speed (*r*^2^ = 0.13). Additionally, we tested within each sub-region and found that the CAN and JIF statistics were similar to the total domain. The WIF, however, had a much weaker relationship between residence time and local wind speed (table 3).

**Table 3.** Residence time versus wind speed.

region	slope	95% CI	*p*-value	*r*^2^
entire domain	−0.18	−0.24 to −0.19	≪0.001	0.13
central canyon	−0.15	−0.25 to −0.06	≪0.001	0.04
Joubin Islands Flank	−0.43	−0.63 to −0.25	≪0.001	0.08
Wauwermans Islands Flank	−0.06	−0.12 to 0.01	0.04	0.01

**Figure 4.**
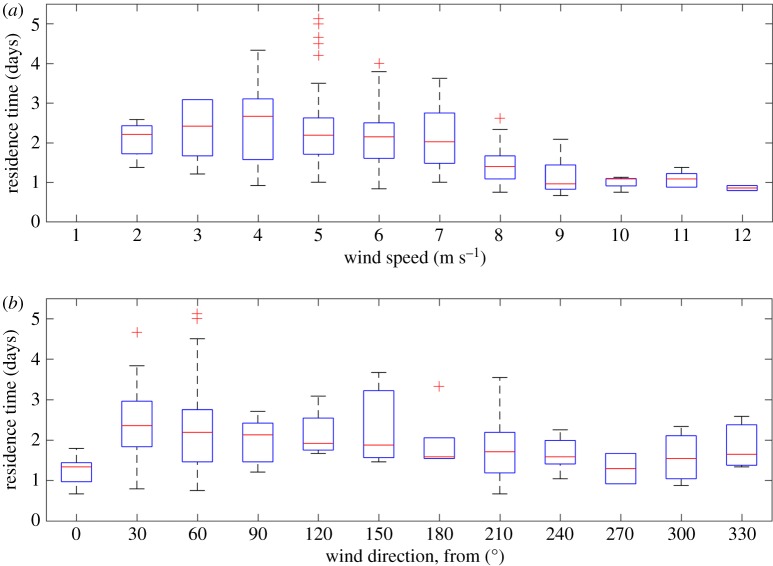
Box-and-whisker plots showing the distribution of residence times binned by (*a*) wind speed and (*b*) wind direction. The sample median, standard deviation and range are shown as a red line, blue box and dashed lines, respectively. Any values that fall outside the data range encompassed by the dashed lines have been indicated with red markers (+).

To further illustrate this, we grouped wind speed into 1 m s^−1^ centred bins. For wind speed less than 8 m s^−1^ there was a much broader distribution of residence times compared to the less frequent high-wind-speed events (figure 4). The same analysis was repeated for wind direction. Here, we binned the wind direction into 30° bins (clockwise from true north). For each bin, we assembled the residence time when winds originated from that direction. Unlike for wind speed, the residence times were highly variable across all wind directions (figure 4). We tested both binned wind speed and binned wind direction in a Tukey multiple comparison test across these bins [30]. Only wind-speed bins 8–11 m s^−1^ showed statistically lower residence times (*p* < 0.01) than wind-speed bins 4–7 m s^−1^. Wind-speed bin 12 m s^−1^ had too few samples to be meaningfully tested. For wind direction, wind from 0° had significantly lower residence times than wind from 30° and 60° (*p* < 0.01). Also, wind from 30° had significantly higher residence times than wind from 210° (*p* < 0.01). Field experience in this region suggests that there is a lot of heterogeneity in wind direction across the domain. While we did have two wind stations in this analysis, it is still insufficient to resolve the local effects of topography on the wind direction over the entire domain. Therefore, the lack of effect on wind direction may be indicative of the poorly resolved wind fields. In general, our analysis suggests that wind speed has a much larger impact on residence time than wind direction.

## 4. Discussion

Currently, canyons along the WAP are thought to be centres of upwelled, nutrient-rich Upper Circumpolar Deep Water (UCDW), which supports local phytoplankton growth and associated food webs [5,12,31]. However, the surface residence times estimated from the HFR currents over Palmer Deep were on average 2 days, with a few outliers as long as 7 days. These residence time estimates based on the highly resolved surface circulation observations are much shorter than the estimated *in situ* water column phytoplankton specific growth rates of 0.01–0.1 d^−1^ (doubling time of 7–70 days) [32]. Even if we relax the definition of residence time to be the time it takes for 90% of the particles to exit the domain, the mean residence time only increases to 4 days, indicating that the longevity of surface particles in this biological hotspot is shorter than reported phytoplankton growth rates. Our surface residence time estimates are also shorter than other reported values along the WAP. Using modelled currents, Piñones *et al*. [19] report surface summer residence times between 6 and 16 days for three hotspots in the central WAP. A similar analysis using the same model, residence time metrics and our HFR domain estimated surface summer residence times for Palmer Deep longer than 10 days [33].

Palmer Deep sits in the middle of the WAP ecosystem, where deeper UCDW is delivered from off the shelf into the canyon below the seasonal mixed layer [13]. Additionally, a coastal current circulating anticlockwise around Anvers Island potentially delivers fresh water into the biological hotspot from the northeast [15]. In general, these currents should advect particles from the west towards the east, consistent with our connectivity results reported in table 2. While our HFR measurements are limited to the surface layer, these high-resolution circulation maps provide an unprecedented look at the mesoscale surface dynamics across the Palmer Deep canyon influencing the surface phytoplankton blooms. During the summer, a buoyant upper layer forms upon warming and surface freshening by ice melt and glacial run-off. These stratified surface layers may be isolated from the canyon bathymetry below and are, therefore, probably driven by different mechanisms than the deeper layers of the canyon. The highest concentrations of chlorophyll, indicative of phytoplankton biomass, are confined to this upper surface layer [25,34]. While barotropic tides influence the entire water column, the surface mixed layer circulation is probably driven primarily by local winds. Our HFR network was able to resolve the dynamics of this surface layer, including the influence of the tides and local winds. These wind-driven processes are typically not well represented in larger-domain numerical models of the WAP shelf.

Our analyses show that surface winds, not local tides, influence local surface residence time on scales of hours to days. Higher wind speeds were shown to reduce surface residence times. These events, while rare in the summer, could have significant influence on the ecology of the Palmer Deep hotspot. Adélie penguin foraging trips near Palmer Station are short (approx. 12 km) compared to other penguin colonies. From 2002–2011, 99.5% of telemetered Adélie penguin locations were located within the contour on figure 1 [6], even though Adélie penguins are capable of very long foraging trips (up to 100 km per day [35]). This suggests that prey fields are not depleted over this relatively short range. One possible explanation for this is that the waters are continually refreshed with new phytoplankton providing food for the Adélie's primary prey, Antarctic krill, thereby sustaining the local krill population and reducing the need for penguins to take longer foraging trips. In addition, Antarctic krill may also be transported into the hotspot, further supplementing the local population.

Antarctic krill undergo diel vertical migration (DVM), leaving the surface waters for deeper layers at night [36]. While DVM has been hypothesized to be a means of predator avoidance [37], it is plausible that this behaviour may also allow Antarctic krill to remain within the Palmer Deep canyon for longer than the typical surface residence time would allow. This is because the deeper layers within canyons exhibit relatively longer residence times (up to 30+ days in the model estimates) [19,33]. The surface layer, on the other hand, supports most of the phytoplankton biomass [25,34] that is targeted by Antarctic krill [38]. Antarctic krill have high daily phytoplankton ingestion rates. Bernard *et al*. [39] showed that summertime ingestion rates of Antarctic krill in the coastal WAP averaged 6.37 µg Chl *a* ind.^−1^ day^−1^ (chlorophyll *a* per individual per day). Abundances of Antarctic krill in aggregations over Palmer Deep are highly variable, with a mean of 87 ind. m^−3^ (standard deviation = 188 ind. m^−3^; K.S. Bernard 2017, unpublished data). Maximum abundances were reached in large, densely packed aggregations (2168 ind. m^−3^; K.S. Bernard 2017, unpublished data). Applying the average ingestion rates of coastal Antarctic krill to these abundance values gives a sense of the grazing impact that these organisms may have on local phytoplankton standing stocks. At an average, grazing rates at the aggregation scale could equate to 0.6 mg Chl *a* m^−3^ day^−1^, with maximum values nearing 13.8 mg Chl *a* m^−3^ day^−1^. A high renewal rate of phytoplankton biomass would be critical to sustaining such high grazing rates. Increased frequency of stronger wind events associated with climate variability (e.g. Southern Annual Mode, El Niño/La Niña) will lead to more frequent flushing events that transport surface particles, including phytoplankton, into and quickly out of the Palmer Deep hotspot.

The passive particle trajectories were consistent with the presence of a persistent coastal current moving east along the southern coast of Anvers Island [15]. In the absence of wind, this current probably drives a mean flow from the canyon east into the Bismarck Strait. Our estimated residence time suggests that the elevated phytoplankton biomass over the central canyon [5] is transported into and out of the hotspot on time scales much shorter than the observed phytoplankton growth rate, suggesting that the canyon may not act as an incubator of phytoplankton productivity as previously suggested [12]. Alternatively, the surface circulation, responsive to variable local winds, may serve more as a conveyor belt delivering phytoplankton biomass produced elsewhere, continually replenishing it for the local Antarctic krill community, which in turn supports numerous top predators. This change in paradigm emphasizes the importance of surface circulation to the delivery of phytoplankton biomass to this biological hotspot.

## Data accessibility

*High-frequency radar surface current data:* The HFR data are archived and accessible through the United States National HF radar archive housed at the National Oceanic and Atmospheric Administration (NOAA) National Data Buoy Center (NDBC): http://hfradar.ndbc.noaa.gov/. Additionally, the post-processed raw and de-tided total vector maps can be accessed via the Rutgers HFR Environmental Research Division Data Access Program (ERRDAPP) Service: http://hfr.marine.rutgers.edu/.
